# Demographic and Clinical Factors Associated with Response to Smallpox Vaccine in Preimmunized Volunteers

**DOI:** 10.1371/journal.pone.0004087

**Published:** 2008-12-31

**Authors:** Philippe Bossi, Frédérick Gay, Imène Fouzai, Béhazine Combadière, Geneviève Brousse, Bénédicte Lebrun-Vignes, Jean-Marc Crance, Brigitte Autran, Daniel Garin, for the IMMUVAR group

**Affiliations:** 1 Department of Infectious Diseases, University Pierre and Marie Curie, Paris VI, Paris, France; 2 Department of Immunology- INSERM U543, University Pierre and Marie Curie, Paris VI, Paris, France; 3 Department of Pharmacology, University Pierre and Marie Curie, Paris VI, Paris, France; 4 Pitié-Salpêtrière Hospital, University Pierre and Marie Curie, Paris VI, Paris, France; 5 Department of Virology, CRSSA Emile-Parde, Grenoble, France; Columbia University, United States of America

## Abstract

**Context:**

In March 2003, the French Ministry of Health implemented a program on preparedness and response to a biological attack using smallpox as weapon. This program included the establishment of a preoutbreak national team that could be revaccinated against smallpox.

**Objective:**

To identify demographic and clinical factors associated with vaccination success defined as the presence of a pustule at the inoculation site at day 8 (days 7–9), with an undiluted vaccinia virus derived from a Lister strain among preimmunized volunteers.

**Volunteers and Methods:**

From March 2003 to November 2006, we have studied prospectively 226 eligible volunteers. Demographic data were recorded for each volunteer (age, sex, number of previously smallpox vaccinations and date of the last vaccination). Smallpox vaccine adverse reactions were diagnosed on the basis of clinical examination performed at days 0, 7, 14, 21 and 28 after revaccination.

**Results:**

A total of 226 volunteers (sex ratio H/F = 2.7) were revaccinated. Median age was 45 years (range: 27–63 yrs). All volunteers completed follow-up. Median number of vaccinations before revaccination was 2 (range: 1–8). The median delay between time of the study and the last vaccination was 29 years (range; 18–60 yrs). Sixty-one volunteers (27%) experienced one (n = 40) or more (n = 21) minor side effects during the 2–14 days after revaccination. Successful vaccination was noted in 216/226 volunteers (95.6%) at day 8 and the median of the pustule diameter was 5 mm (range: 1–20 mm). Size of the pustule at day 8 was correlated with age (p = 0.03) and with the presence of axillary adenopathy after revaccination (p = 0.007). Sex, number of prior vaccinations, delay between the last vaccination and revaccination, and local or systemic side effects with the exception of axillary adenopathy, were not correlated with the size of the pustule at day 8.

**Conclusions:**

Previously vaccinated volunteers can be successfully revaccinated with the Lister strain.

## Introduction

The events of 2001 in the United States had worldwide repercussions on the awareness of bioterrorism as well as on the development of plans to counteract bioterrorism amongst many countries. Governments and international entities with responsibilities related to maintenance of peace, security, safety and health protection reviewed urgently their political, economic, diplomatic, military and legal means to face up to such attacks and embarked upon major efforts to increase their preparedness. Individual nation states have developed plans to fight against bioterrorism and have united in order to resist this threat. More than 150 pathogens have been reported as potential agents for bioterrorism [1]. Among them, smallpox represents a high threat [2]–[4]. It was declared eradicated worldwide by the World Health Organisation in 1979 following a smallpox-eradication campaign begun in 1958 and intensified in 1967, and the last case of endemic smallpox occurred in Somalia in 1977 [5]. Throughout world, vaccination against smallpox has been discontinued since about 1982. In France, routine childhood immunization using the Lister strain ceased in 1979, and reimmunization of healthcare workers in 1984 [6]. This means, that a large population is susceptible, and any new case of smallpox would have to be the result of human accidental or deliberate release, could be associated to a major epidemic.

In March 2003, the French Ministry of Health implemented a program on preparedness and response to a biological attack using smallpox as weapon [7]. This program included the establishment of a preoutbreak national team that could be revaccinated against smallpox and can be called upon to investigate and manage initial suspected or confirmed cases of smallpox in France. People, who were selected to this team, were eligible volunteers, had received at least one dose of smallpox vaccine in the past and have no contraindication to a reimminization. This program used the Lister strain (Pourquier® vaccine) which received French Health Products Safety Agency (AFSSAPS) licensure for this exclusive purpose.

The objective of this study was to identify demographic and clinical factors associated with vaccination success, defined as the presence of a pustule at the inoculation site eight days post vaccination and measured by the size of the pustule among 226 eligible preimmunized volunteers.

## Methods

From March 2003 to November 2006, we have studied prospectively 226 eligible volunteers selected for the French national team against smallpox. They have received at least one dose of smallpox vaccine in the past, without major side effects, and have evidence of vaccine “take” as evidenced by the presence of a scar resulting from previous smallpox vaccination or notification on a notebook vaccination. Reimmunization for this team was performed with the Lister strain (Pourquier® vaccine). Screening to identify and exclude subjects with contraindications for them or household close contacts to smallpox immunization was performed (table 1). An HIV test was performed two weeks before vaccination. A urine pregnancy test on the day scheduled for vaccination was also performed for women of childbearing age. Written informed consent was obtained from all volunteers. French Health Products Safety Agency (AFSSAPS) approved this study.

**Table 1:** Contraindications against vaccination for volunteers and household contacts (7).

• History or presence of eczema or atopic dermatitis
• Other active acute, chronic or exfoliative skin conditions that disrupt the epidermis
• Conditions associated with immunosuppression (cancer, HIV, chemotherapy, radiotherapy, transplantation, autoimmune conditions, immunosuppressive medications…)
• Pregnancy, desire to become pregnant in the month following the revaccination or breastfeeding
• Having children <1 year
• Allergy to any component of the vaccine (polymyxin B, streptomycin, tetracycline or neomycin)
• Neurological disease
• Taking ocular steroid medication
• Heart disease
• Fever within 8 days before revaccination

There is currently two licensed smallpox vaccine in France using the Lister strain. There are approximately 72 millions doses of vaccine potentially available. The vaccine used (Pourquier® vaccine) contains live unattenuated vaccinia virus derived from the Lister strain which produces cross immunity against variola major and minor. The titer of vaccinia virus was 10^7.7^ pfu/mL. This lyophilized vaccine, product prepared from calf lymph, was reconstituted by adding sterile diluent to the powder and administered into the dermis by using the multiple-puncture technique with a presterilized bifurcated needle (10–15 punctures in an area of about 5 mm in diameter as recommended for revaccination over the insertion of the left deltoid muscle). The same physician performed all the vaccinations to exclude technical problems associated with the vaccination procedure. The vaccination site was covered with gauze in combination with a semipermeable membrane. Dressings were changed and the vaccination sites were assessed every seven days (d0–d28) until the lesions dried and an eschar formed. Following revaccination, vaccination success was measured by the development of a vaccination site take, defined as the presence of a pustule at the inoculation site 7 to 9 days post vaccination.

Demographic data were recorded for each volunteer (age, sex, number of previously smallpox vaccinations and date of the last vaccination). There were no restrictions on work activities following revaccination. Smallpox vaccine adverse reactions were diagnosed on the basis of clinical examination performed at days 0, 7, 14, 21 and 28 after revaccination. Volunteers were also questioned at each follow-up visit for the presence of any vaccine-related adverse events such as local and systemic symptoms for at least 4 weeks after revaccination. Fever was defined as temperature ≥37.8°C.

The quantitative variables (i.e.: age, size of the pustule, delay between the vaccination and the revaccination) are not normally distributed. By consequence, the non parametric tests using exact calculation rather than asymptotic ones have been applied by using StatXact® software version 6 (Cytel Studio). Without any certitude regarding the hypothetic direction of the relation, all tests have been interpreted based on a two-tails p-value. The α risk has been chosen at 5%. The Bonferoni correction has been taken into account according to the number of tests done. The quantitative variables have been crossed by using the Mann-Whitney exact test. The Spearman rank correlation test has been used to test the correlation between quantitative variables.

## Results

A total of 226 volunteers (sex ration H/F = 2.70) were revaccinated and included in this study (table 2). Median age of the cohort was 45 years (range: 27–63 years). All volunteers completed follow-up. All volunteers have been vaccinated against smallpox for the first time before one year old. Median number of vaccinations before revaccination was 2 (range: 1–8). Among volunteers, 45 (19.9%) received only one vaccination in the past. The median delay between time of the study and the last vaccination was 29 years (range: 18–60 years).

**Table 2:** Characteristics of the volunteers before revaccination.

Characteristics	Results
Number of volunteers	226
Sex ratio (M/F)	2.7 (165/61)
Median age (range) yr	45 (27–63)
Median number of prior vaccination (range)	2 (1–8)
Median delay between the last vaccination yr (range)	29 (18–60)

Among the 226 volunteers, 61 (27%) experienced one (n = 40) or more (n = 21) minor side effects during the 2–14 days after revaccination. No side effect was reported during the third and the fourth week after revaccination. No serious adverse effect was notified and no adverse event was reported in their household close contacts. Moreover, no cardiac complication following revaccination was reported in this study. Local symptoms were: local pruritis (n = 17) (8%), axillary lymphadenopathy (n = 7) (3%), and large vaccination reaction (robust take) >7.5 cm in diameter (n = 2) (1%), and systemic symptoms were: fever >37.8°C (n = 27) (12%), fatigue (n = 14) (6%), headache (n = 6) (3%), myalgia (n = 6) (3%), nausea (n = 2) (1%), diarrhea (n = 2) (1%) and cough (n = 2) (1%) (Table 3).

**Table 3:** Local and systemic symptoms among the 226 volunteers after the revaccination*.

Adverse events	n (%)
fever >37.8°C	27 (12)
Fatigue	14 (6)
local pruritis	17 (8)
axillary lymphadenopathy	7 (3)
headache	6 (3)
myalgia	6 (3)
nausea	2 (1)
diarrhea	2 (1)
cough	2 (1)
large vaccination reaction (diameter >7.5 cm)**	2 (1)

* 61 volunteers (27%) experienced one (n = 40) or more (n = 21) side effects.

** ref (30).

Successful vaccination was noted in 216/226 volunteers (95.6%) at day 8 (days 7–9). Median of the pustule diameter in these 216 volunteers, at day 8 after revaccination was 5 mm (range: 1–22 mm): median age was 45.5 years (range: 27–63 years) and median delay between time of the study and the last vaccination was 29 years (range: 18–60 years). Among volunteers, 10 did not develop any clinical take (pustule diameter = 0) at the vaccination site (median age was 41 years (range: 33–49 years) and median delay between time of the study and the last vaccination was 33.5 years (range: 20–45 years)). We did not find any difference between these two groups of volunteers with or without pustule.

A larger size of the pustule at day 8 was statistically correlated with a greater age (p = 0.03) and with the presence of axillary adenopathy after revaccination (p = 0.007). Sex, number of prior vaccinations, delay between the last vaccination and revaccination, and local or systemic side effects with the exception of axillary adenopathy, were not associated with the size of the pustule at day 8.

## Discussion

The data of this study shows that volunteers previously immunized with smallpox vaccine are successfully revaccinated with an undiluted Lister strain.

Successful vaccination was noted in 216 of the 226 volunteers (95.6%). This result is in accordance with other studies which used an undiluted Lister strain [8], [9]. Size of the pustule was significantly correlated with age and presence of axillary adenopathy after revaccination. As reported in another study, sex, number of prior immunizations and delay between the last vaccination and revaccination were not correlated with the size of the pustule at day 8 [10].

It has been usually reported that compared with vaccinia-naïve subjects, prevaccinated patients had significantly smaller pustule lesion [11]. The fact that a larger size of the pustule was statistically correlated in our study with a greater age, suggests that some older prevaccinated volunteers are similar to vaccinia-naïve individuals who do not have any immunity to smallpox. This can be due to loss of immune function with aging. However, this loss of immune function, is not correlated with number of prior immunizations and delay between the last vaccination and revaccination: both CD4+ and CD8+ cells specific for vaccinia virus have been found to persist for up to 75 years after the last immunization [12]–[14]. Moreover, persistence of humoral immunity to smallpox has been reported in patients up to 60 years after the last immunization [15].

In the literature, local lymphadenopathy is reported in 7 to 88% of cases [16]. But usually, and whatever the vaccine used, it has been reported that more naive subjects presented regional lymphadenopathy than non-naive subjects [17], [18]. This could be the fact that vaccinia-naïve volunteers shed virus from the vaccination site 2 to 6 days longer and had significantly higher peak mean viral titers when compared with prevaccinated volunteers [8], [11].

It has been also reported that the 1∶5 and 1∶10 dilutions of smallpox vaccine in adults who had not been previously immunized, were associated with a smaller incidence of adenopathy than those reported in naïve subjects given undiluted vaccine [19], [20]. This is in accordance with our findings, suggesting that volunteers who have a larger pustule due to an increased local inflammation have an increase incidence of regional lymphadenopathy.

In our study, only 61 patients (27%) experienced at least one minor side effect during the 2–14 days after revaccination with the Lister strain. These side effects included only moderate local and/or systemic adverse events. No serious adverse effect, such as postvaccinal encephalitis, progressive vaccinia, eczema vaccinatum, generalized vaccinia, inadvertent inoculation, cardiac complication or death was notified in the volunteers and their household close contacts [21]–[25]. Local and systemic symptoms are quite common with this live viral vaccine, consistent with the presence of an acute viral illness. However, with the exception of fever, all the symptoms observed in our study were less frequent than those reported in vaccinia naïve volunteers vaccinated with a lyophilized form (Dryvax, Wyeth Laboratories, Marietta, Pa.) and a frozen preparation (Aventis Pasteur, Swift-water, Pa.), live-virus vaccines, both derived from the New York City Board of Health vaccinia strain, even at diluted doses [17], [19], [20], [26]. However, frequency of symptoms were in accordance with those reported in non-naïve volunteers in other studies, including the Lister strain [8], [17]–[19].The fact that fewer adverse reactions are observed in non-naïve patients when compared with events in vaccinia-naïve participants are probably due to immunologic memory. This hypothesis is supported by the differences between previously vaccinated subjects and vaccinia-naïve subjects in local and systemic symptoms and signs and the quantify of viral shedding [8].

Previously vaccinated volunteers can be successfully revaccinated with the Lister strain. Large studies are needed to analyse second and third generations vaccines [27]–[29].
